# OSPACS: Ultrasound image management system

**DOI:** 10.1186/1751-0473-3-11

**Published:** 2008-06-20

**Authors:** Will Stott, Andy Ryan, Ian J Jacobs, Usha Menon, Conrad Bessant, Christopher Jones

**Affiliations:** 1 Bioinformatics Group, Cranfield University, Bedfordshire, MK43 0AL, UK; 2 Gynaecological Cancer Research Centre, UCL EGA Institute for Women's Health, University College London, Huntley Street, London WC1E 6DH, UK; 3 Gynaecological Cancer Research Laboratories, UCL EGA Institute for Women's Health, University College London, Paul O'Gorman Building, Gower Street, London WC1E 6BT, UK

## Abstract

**Background:**

Ultrasound scanning uses the medical imaging format, DICOM, for electronically storing the images and data associated with a particular scan. Large health care facilities typically use a picture archiving and communication system (PACS) for storing and retrieving such images. However, these systems are usually not suitable for managing large collections of anonymized ultrasound images gathered during a clinical screening trial.

**Results:**

We have developed a system enabling the accurate archiving and management of ultrasound images gathered during a clinical screening trial. It is based upon a Windows application utilizing an open-source DICOM image viewer and a relational database. The system automates the bulk import of DICOM files from removable media by cross-validating the patient information against an external database, anonymizing the data as well as the image, and then storing the contents of the file as a field in a database record. These image records may then be retrieved from the database and presented in a tree-view control so that the user can select particular images for display in a DICOM viewer or export them to external media.

**Conclusion:**

This system provides error-free automation of ultrasound image archiving and management, suitable for use in a clinical trial. An open-source project has been established to promote continued development of the system.

## Background

Medical sonography (ultrasonography) uses ultrasound to provide real-time images of soft tissues, internal organs and the fetus *in utero*. Because medical sonography is non-invasive and generally considered to have no harmful side effects, it has seen increasing use for a variety of diagnostic purposes in recent years. One of the most common applications of ultrasound imaging is in routine obstetric care, assessing the stage and status of pregnancy and the health and development of the fetus. Other applications include the imaging of most of the internal organs, muscles, ligaments and tendons.

DICOM 3.0 (Digital Imaging and Communications in Medicine) is a standard describing the handling, transfer and storage of medical imaging data, including ultrasound scans [1]. A DICOM data object (or data set) combines a medical image in one of several standards (either still, or video) with patient information and other scan data. The linked storage of these data is an important feature of the standard, ensuring that the descriptive patient data are always associated with the correct medical image. Most modern health care facilities store these DICOM objects in a picture archiving and communication system (PACS), allowing ultrasound scan records to be managed in the same way as other types of medical images.

A considerable amount of work is currently being undertaken to evaluate the use of ultrasound in various new diagnostic procedures. The clinical trial of a new ultrasound procedure generates significant quantities of scan data that typically require cross-comparison and peer assessment. However, the nature of clinical trials often precludes the storage of data alongside patient records in an existing PACS, and the purchase of a PACS for the limited use of a trial is usually not cost effective. As a result the ultrasound scans generated by clinical trials are not always stored in a way that facilitates their management and future retrieval, and our own experience of this issue was the incentive for this project; we had attempted to manage large numbers of ultrasound images using software supplied with the ultrasound machine, only to discover that it became unmanageable when the number of images exceeded a certain threshold.

In order to satisfy the need for the management of ultrasound images without incurring the effort and expense of setting-up a commercial PACS, we have developed OSPACS. This system is based on a simple Windows application utilizing an open-source DICOM image viewer and a relational database. OSPACS is currently being used to manage the UKCTOCS (United Kingdom Collaborative Trial for Ovarian Cancer Screening) [2] ultrasound archive, providing error-free automation of ultrasound image archiving and management. An open-source project has been established in order to promote the continued development of OSPACS for UKCTOCS as well as other clinical trials.

## Implementation

OSPACS is implemented using traditional client-server architecture, such that the client is comprised of a Windows Forms application (osImageManagerApp.exe), which accesses a server hosting the image database.

### Software Development and Design

Development of OSPACS followed an Agile approach [3] inspired by Extreme Programming (XP) [4], utilizing practices such as Real Customer Involvement, Incremental Deployment, Incremental Design and Test-First Programming. This allowed the system to evolve in an incremental way through a series of iterations that were driven by the need to frequently deliver valuable software that satisfied the end-user (customer).

The Incremental Design practice requires design to be performed everyday instead of being confined to a particular phase during the project (as is the case in a Waterfall process) or during the iteration (as is the case in Rational Unified Process). In practical terms this means following the Test-First Programming practice so that the design evolves in a bottom-up fashion. However, Agile Modeling techniques were employed during the creation of the initial architecture and before important design decisions were taken, so the design of OSPACS was actually produced by a combination of bottom-up and top-down approaches.

### Image Database

We have implemented the image database using the Microsoft SQLServer database management system (DBMS) [5]. Because this is a commercial product, the OSPACS setup program includes the option of installing the Express Edition of SQLServer, which is available free of charge from the Microsoft website [5]. SQLServer Express is functionally identical to the full commercial product in respect of OSPACS requirements, but the database size is limited to 4GB. While this constraint will significantly limit the number of ultrasound records that can be stored, the use of SQLServer Express will allow the system to be evaluated adequately.

During its design we attempted to follow the maxim of developing "the simplest thing that could possibly work" [6]. Consequently there is just one table with eleven fields, the most important of which is the DicomFile field (see Table 1). This field is an Image data type (a large binary object, or BLOB) and contains the entire contents of the DICOM object as binary data. However, as the application software only accesses the database through a set of stored procedures, it should be possible to extend this schema relatively easily.

**Table 1:** Structure of the Image database table

	**Column Name**	**Condensed Type**	**Nullable**
*PK*	ImageID	uniqueidentifier	No
	CaptureDate	datetime	Yes
	StoredDate	datetime	Yes
	StoredBy	varchar(50)	Yes
	DiskRef	varchar(50)	Yes
	VolunteerRef	int	Yes
	DicomRef	varchar(64)	Yes
	DicomFile	image	Yes
	DicomFileSize	int	Yes
	MaskFile	varchar(255)	Yes
	Flags	varbinary(10)	Yes

*PK *indicates the Primary Key.

### ezDICOM Component

The ezDICOM component is free, open source software that can be used to view a wide range of medical images including the DICOM standard and proprietary formats [7]. It has a good reputation for being mature, stable software and has been successfully used in a number of other Medical Imaging applications. OSPACS uses the ezDICOM ActiveX control under the BSD open source license.

### Software Development Tools and Libraries

The OSPACS software was developed in C# using Visual Studio 2005 tools (Microsoft) and it includes a collection of automated unit tests which cover more than 95% of the code base. The user interface layer is implemented as a Windows Forms application and uses the .NET 2.0 Framework Class Library (FCL) [5] to provide the main window, dialog boxes, associated controls, *etc*. Non-functional requirements, such as logging and error handling, were implemented using the third-party library MxToolbox [8]. Automated functional tests were developed using the Framework for Integrated Test (FIT) library [9].

## Results and Discussion

### Installation

Installation of the client part of OSPACS is achieved by running the installation program (setup.exe) downloaded from the OSPACS open-source site [10]. This installation program can also install Microsoft SQLServer Express and the Microsoft .NET 2.0 Framework Library, if required. Once the basic installation has been performed the system's on-line help explains the steps required to set-up the databases.

The standard installation process will install an application called osImageManager and create both an image database and a test patient database on the local computer. The FIT automated functional tests can then be run from the application's 'Database Admin' dialog box in order to validate the system within the context of the client PC. For the system to be used in a production context it is necessary only to change the configuration of the database sources to the required servers, and then re-run the scripts from the application's Database Admin dialog box in order to create any production image database that might be required. In this way the installation of OSPACS is made simple, repeatable and reliable.

### Image import

Ultrasound images in the DICOM image format can be imported as files from removable media, and information from the DICOM image header cross-checked against an external database. The current implementation of OSPACS is specific to UKCTOCS, and patient details from the DICOM header are compared to specific data fields in the patient information tables of the main UKCTOCS database. Where information in the DICOM header does not agree with a UKCTOCS database entry, the image is flagged and options to correct data in the DICOM header are provided. Following a successful validation of data in the DICOM header, the relevant information is extracted and used to populate fields in a new image database record.

Patient anonymity is protected by removing all patient-identifying information from the DICOM header data, and also by masking parts of the image data itself before populating the database record. The region of the image containing the patient identifier does not vary in scans from similar hardware, so a defined region of the image is simply over-written with white pixels.

### Viewing images

**Figure 1:**
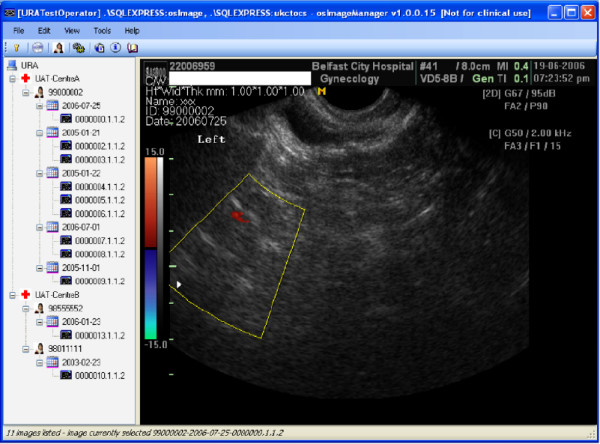
**The osImageManager application**. Screenshot of the osImageManager application, showing the treeview control and DICOM viewing window.

Once DICOM objects have been imported into the image database they can be readily retrieved by running a standard query using values entered into a dialog box opened from osImageManager's View menu. When the dialog box is closed the records in the database matching the query value are displayed in a standard Windows treeview control, arranged in a heirarchy of scanning centre, patient ID, scan date and images (see Figure 1). Selection of an image item in the treeview displays the corresponding image in a viewing window (see Figure 1). Images can be retrieved either by reference number of the trial volunteer, or the reference number of the removable media entered during the import process.

### Image export

Selected images in the treewiew can easily be saved to removable media in their original file format. While this process does not create a DICOMDIR file (defined by the DICOM standard, a directory that indexes and describes all of the DICOM files that are stored on the media) and hence is not fully compliant with the DICOM standard, these files can be opened and viewed by any DICOM viewing application.

## Conclusion

OSPACS has been successfully implemented within the Gynaecological Cancer Research Centre, where it is being used to manage ultrasound images collected as part of the United Kingdom Collaborative Trial of Ovarian Cancer Screening (UKCTOCS). During the course of the trial it is expected that UKCTOCS will accumulate approximately 1,500,000 ultrasound images, from 300,000 examinations, collected by 13 regional centres throughout the UK. OSPACS is currently being used to automate the import of scans from the regional centres, helping to resolve patient identifiers with the central UKCTOCS database, and to export groups of images to removable media for external review. There are currently ~200,000 ultrasound images stored in the UKCTOCS ultrasound record archive (UKCTOCS URA), which translates to ~150 Gb of disk space. In summary, OSPACS has been used to create a usable ultrasound record archive, ensuring the integrity and helping to realise the research potential of a valuable and irreplaceable resource.

Future development of the OSPACS project will include the generation of a valid DICOMDIR file during image export to removable media, enabling better integration of OSPACS with other DICOM and PACS applications. Also, support for other database management systems besides Microsoft SQLServer, such as the widely used open source (free) platforms MySQL and PostgreSQL, will be implemented. Finally, the implementation of lossless compression of the DICOM images in order to reduce the overall size of the database and increase the speed of database transactions is planned. The creation of an open source project will facilitate this continued development, and provide the opportunity for others to modify and extend the functionality of OSPACS.

## Availability and requirements

• **Project name: **OSPACS

• **Project home page: **

• **Operating system(s): **Windows XP

• **Programming language: **C#

• **Other requirements: **Microsoft SQLServer (or SQLServer Express)

• **License: **Cranfield Open-Source License

• **Any restrictions to use by non-academics: **License required

## List of abbreviations

PACS: Picture Archiving and Communication System; DICOM: Digital Imaging and Communications in Medicine; UKCTOCS: The United Kingdom Collaborative Trial of Ovarian Cancer Screening.

## Competing interests

The authors declare that they have no competing interests.

## Authors' contributions

WS designed, wrote and implemented all source code and helped in the preparation of the manuscript, AR specified system requirements, and tested each release of the system on the UKCTOCS Ultrasound Record Archive, IJ provided infrastructure and funding support, UM contributed to the design of the project, CB supervised the work of WS towards an MSc in Bioinformatics at Cranfield University, CJ conceived and coordinated the project, and was responsible for preparation of the manuscript. All authors read and approved the final manuscript.
